# Early Diagnosis of Canine Hip Laxity: Correlation between Clinical Orthopedic Examinations and the FCI Scoring Method in a Closed Cohort of Rottweilers

**DOI:** 10.3390/ani11020416

**Published:** 2021-02-06

**Authors:** Britta Vidoni, Veronika Bauer, Barbara Bockstahler, Michaela Gumpenberger, Alexander Tichy, Masoud Aghapour

**Affiliations:** 1 Small Animal Surgery, University Clinic for Small Animals, Department for Companion Animals and Horses, University of Veterinary Medicine, 1210 Vienna, Austria; Britta.Vidoni@vetmeduni.ac.at; 2 University Clinic for Small Animals, Department for Companion Animals and Horses, University of Veterinary Medicine, 1210 Vienna, Austria; Veronika.Bauer@vetmeduni.ac.at; 3 Section of Physical Therapy, Small Animal Surgery, University Clinic for Small Animals, Department for Companion Animals and Horses, University of Veterinary Medicine, 1210 Vienna, Austria; Barbara.Bockstahler@vetmeduni.ac.at; 4 Diagnostic Imaging, University Clinic for Small Animals, Department for Companion Animals and Horses, University of Veterinary Medicine, 1210 Vienna, Austria; Michaela.Gumpenberger@vetmeduni.ac.at; 5 Department for Biomedical Science, Platform Bioinformatics and Biostatistics, University of Veterinary Medicine, 1210 Vienna, Austria; Alexander.Tichy@vetmeduni.ac.at

**Keywords:** hip laxity, canine hip dysplasia, Ortolani, reduction angle

## Abstract

**Simple Summary:**

Canine hip dysplasia is one of the most frequently occurring orthopedic diseases in dogs, and hip laxity is the primary sign of this disease. The early diagnosis of hip laxity in puppies would make veterinarians capable of planning preventative procedures to treat the disease or reduce the severity of disease at older ages. These procedures would improve the quality of the life of the dogs and reduce treatment costs. Furthermore, because of the importance of genetics in this disease, dogs with a risk of developing canine hip dysplasia in the future could be excluded from breeding programs. Therefore, the early diagnosis of canine hip laxity, as well as the selection of proper diagnostic methods, are of great importance in small animal orthopedics.

**Abstract:**

Canine hip dysplasia is a multifactorial disorder characterized by hip laxity and osteoarthritis. The early diagnosis of hip laxity is an important topic in small animal orthopedics. This study aimed to evaluate the correlation between clinical orthopedic examinations and the Fédération Cynologique Internationale (FCI) scoring method. Thirty purebred Rottweilers were examined at approximately four (20 ± 2 weeks), eight (35 ± 2 weeks), and twelve months of age (54 ± 1 weeks), respectively. The Ortolani, Barlow, and Bardens tests and reduction/subluxation angle measurements were performed at each time. FCI scoring was conducted at the third examination time. Positive correlations were recorded between the reduction angle and Ortolani test, reduction angle and FCI score, and Ortolani test and FCI score for the second and third examination dates. No correlation was observed between the subluxation angle and other methods. Despite previous studies reporting 16–20 weeks as the earliest age for diagnosing hip laxity in dogs, in our study, early diagnosis was possible from the age of 35 ± 2 weeks. This difference might originate from the small sample size, low number of the dogs with severe grades of laxity, and breed differences.

## 1. Introduction

Canine hip dysplasia (CHD) is a complex, polygenic, and multifactorial disorder characterized by hip joint laxity and osteoarthritis [1,2]. Hip laxity is a primary sign of hip dysplasia and a major risk factor for the development of coxofemoral osteoarthritis. According to the scientific literature, the main underlying reason for hip joint laxity is still under investigation, but parameters such as heredity, body size and body mass, fat intake, rapid growth, ossification of the hip joint, pelvic shape, the skeletal structure of the hind limbs, an increased amount of synovial fluid in the hip joint, and the hormonal influence of relaxin have been reported as predisposing factors in the literature [3,4,5]. Subluxation of the femoral head during the gait cycle causes micro-fractures in the subchondral bone due to an abnormal distribution of forces. Therefore, in the long run, this process leads to osteophyte formations and osteoarthritis [6]. Previous studies have shown a direct relationship between the severity of joint laxity and the presumption of osteoarthritis formation. The risk of osteoarthritis rises with the degree of joint laxity [7,8,9].

Two mechanisms are responsible for the development of hip laxity, including malformation of the acetabulum or femoral head and developmental disorders of the capitis femoris ligament. The os coxae quartum (OCQ) shapes the proximal part (roof) of the acetabulum and plays an important role regarding the shape, contour, and depth of the acetabulum. This bone raises forwards and backwards from the joint cartilage and reaches the cranio-lateral acetabular rim [10]. This T-shaped bone can be recognized from the beginning of ossification at 12 weeks of age in radiographs. At the end of ossification at 20 weeks of age, this bone fuses with the other parts of the acetabulum [10]. Ossification disorders such as cranial hypoplasia of the OCQ lead to a flat acetabulum and joint instability [10]. Degeneration of the femoral head ligament (ligamentum capitis femoris) can be noticed from 10 weeks of age due to hip laxity. In this disorder, the acetabulum is correctly ossified and reaches its physiological depth. This joint laxity is most likely the result of dysplasia of the joint’s soft tissue, such as the ligamentum capitis femoris and the capsule itself. Based on the first occurrence of each mechanism, the laxity is divided into the primary osseous dysplasia (dysplasia of the acetabulum or of the femoral head) and primary soft tissue dysplasia (dysplasia of the ligamentum capitis femoris and joint capsule) [10].

An evaluation of the hip joint is performed using special orthopedic examinations and radiographic assessments followed by preventive surgeries in the case of increased laxity in puppies or young dogs, such as juvenile pubic symphysiodesis (JPS) in mild to moderate laxity and double or triple pelvic osteotomies (DPO or TPO, respectively) in higher grades of joint laxity. Conventional therapeutic surgery in adult dogs suffering from hip dysplasia consists of femoral head and neck ostectomy and total hip arthroplasty.

The Barlow, Ortolani, and Bardens tests are early diagnostic orthopedic examinations for hip dysplasia [11,12,13]. The mechanisms of these qualitative examinations are based on the instability of the hip joint. All three tests can be demonstrated with existing hip joint laxity, and they may also presuppose the occurrence of hip joint laxity. The Barlow test applies to luxation/subluxation of the femoral head, which is triggered manually by the examiner [11]. The Ortolani test consists of sliding the femoral head back into the acetabulum in a luxated/subluxated hip and can be performed in dorsal or lateral recumbency [12]. The Ortolani maneuver can be performed, at the earliest, from the age of 7–8 weeks in dorsal or lateral recumbency, and a 92% sensitivity was reported for this test in the literature [14,15]. The Bardens test is based on horizontal-lateral mobility of the trochanter major with respect to the tuber sacrale and tuber ischiadicum [13]. A combination of the goniometric measurements of coxofemoral alignments as quantitative measurement methods, along with qualitative orthopedic examinations, can provide further information about the status of the hip joint and severity of the hip luxation. Goniometric examinations consist of measurements of the reduction angle (RA) and subluxation angle (SA) [16]. The RA is the degree of abduction of the femur, at which the femoral head can be reduced or relocated into the acetabulum. The existence of the RA is an indication of the expansion or dilatation of the joint capsule. Therefore, no RA should exist in sound hips; however, in dogs with chronic hip laxity, the RA might be negative due to the fibrotic restriction of the joint capsule [14,17]. As reported in the literature, the RA correlates with the degree of joint laxity [17,18]. An RA ranging from 10° to 25° is considered mild joint laxity and an RA ≥ 25° is considered to be high-grade joint laxity [17]. The likelihood of hip dysplasia or coxarthrosis increases with the size of the RA; for hips with an RA of 15° or higher, 85% osteoarthritis can be predicted [19].

The SA is the degree of limb adduction at which the femoral head can be subluxated. The SA indicates the flattening of the acetabulum. Therefore, no subluxation angle should be detected in a healthy hip joint. Up to ±5° of SA might be seen in dogs with mild joint laxity [17]. In dogs with chronic hip laxity, no SA might be seen due to the fibrotic restriction of the joint [14,20]. The SA corresponds to the shape of the acetabular rim and the dorsal acetabular rim angle (DAR angle), which is provided by DAR view radiographs [14,17,21]. The DAR angle is defined by the inclination of the dorsal acetabular margin with respect to the line traced perpendicularly to the major axis of the pelvis [22]. In sound dogs, the lateral section of the dorsal acetabular rim is sharp, the femoral head is located deep in the acetabular cavity, and the DAR angle is less than 7.5° [21,22].

Radiography has been used as a gold standard for a long time, to assess the condition of the hip joint, and different performance methods have been developed over the years. The guidelines of the Fédération Cynologique Internationale (FCI) are used in many European countries, Asia, and parts of South America [23]. According to these guidelines, standard ventrodorsal radiographs are taken at the age of 12 or 18 months (reaching skeletal maturity) to evaluate the degree of the Norberg angle (NA), which offers information about the position of the center of the femoral head relative to the craniolateral acetabular margin. In normal hips, the NA is reported to be about 105°, whereas in subluxated femoral heads, a much smaller NA is expected [24]. The main focus of the FCI method is primarily the diagnosis of hip joint laxity and secondarily the evaluation of degenerative joint disease, or osteoarthritis. The severity of laxity is categorized into five grades, from HD A (no sign of hip dysplasia) to HD E (severe hip dysplasia) in this method [24]. This grading method is based on the measurement of the NA, the degree of subluxation/luxation of the center of the femoral head relative to the acetabulum in a standard hip-extended view, and the presence of degenerative joint disease [24].

The Pennsylvania Hip Improvement Program (PennHIP) is another method developed by the University of Pennsylvania. This method is based on three radiographic assessments, including the FCI standard hip-extended view, a compression view, and a distraction view. The distraction index (DI) is the ratio of the distance between the center of the femoral head, the center of the acetabulum, and the radius of the femoral head. The DI is a number between 0 and 1, where a higher DI increases the risk of hip laxity. The possibility of the occurrence of hip dysplasia in dogs with DI > 0.6 is high and DI ≤ 0.3 is low [7,25].

In previous studies, different protocols have been reported to measure hip joint alignments, and different orthopedic and radiographic examinations have been investigated to predict the occurrence of hip joint laxity in puppies [5,7,9,10,11,12,13,16,17,26]. In the current study, the correlation between orthopedic examinations, including Ortolani, Barlow, and Bardens tests, SA, RA, and the FCI scoring method was assessed in a closed cohort of Rottweilers. The aims of this study were: (1) to report the descriptive statistics of the Ortolani, Barlow, and Bardens tests in juvenile purebred Rottweilers at 4, 8, and 12 months of age; (2) to investigate whether there is a statistical difference between Ortolani findings in dorsal and lateral recumbency; (3) to investigate whether there is a statistical difference between the Barlow sign in dorsal and lateral recumbency; (4) to report the mean and standard deviation for measured RA and SA; (5) to assess the dogs at the age of 12 months according to the FCI guidelines; (6) to investigate whether the measured RA correlates with results of the Ortolani sign; (7) to investigate if there is a positive correlation between the degree of RA/SA and radiographic findings according to the FCI guidelines; and finally (8) to investigate whether the Ortolani findings in dorsal recumbency correlate with the results of the FCI scoring method.

We hypothesized that: (1) there is a statistical difference between Ortolani findings in dorsal and lateral recumbency; (2) there is a statistical difference between Barlow findings in dorsal and lateral recumbency; (3) RA correlates with the results of the Ortolani sign; and (4) final FCI grade can be predicted by the results of the RA/SA and Ortolani findings on dorsal recumbency at an early age of the dog.

## 2. Materials and Methods

### 2.1. Approval and Consent

This study was discussed and approved by the institutional ethics and animal welfare committee in accordance with good scientific practice guidelines and national legislation (ETK-17/12/97/2015). Thirty purebred Rottweilers of the Austrian Armed Forces were evaluated three times in this study. The examinations were performed at mean ages of 20 ± 2 weeks (M1), 35 ± 2 weeks (M2), and 54 ± 1 weeks (M3), respectively. All of the dogs underwent a general clinical examination, primarily while awake, to evaluate their health status, and an orthopedic examination to detect any existing lameness or swelling and asymmetries of the musculoskeletal system. All examinations were performed by the same investigator (B.V.). During the examinations, the declared age was checked by the presented breeding documents of each dog. The weight of each dog was also recorded.

### 2.2. Anesthesia

After clinical and orthopedic examinations of the awake dogs, all further orthopedic examinations were performed under general anesthesia to prevent possible pain and achieve complete relaxation of the musculature because of the importance of the correct positioning in radiographic studies. The dogs were premedicated with Medetomidine (0.01–0.02 mg/kg, IV). The induction and maintenance of the anesthesia was done with Propofol (1–5 mg/kg, IV, and 0.2 mg/kg/min, IV, respectively)

### 2.3. Orthopedic Hip Examinations under General Anesthesia

#### 2.3.1. Barlow Maneuver

The Barlow maneuver was performed in lateral and dorsal recumbency. The test was conducted with the adducted femur. The pressure was applied to the femur in the proximal direction during adduction. Luxation/subluxation of the femoral head was termed Barlow positive. Barlow positive hips were confirmed by the Ortolani maneuver by reduction of the hip joint. The Barlow maneuver can be considered a prerequisite of the Ortolani maneuver.

#### 2.3.2. Ortolani Maneuver

The Ortolani test was conducted with an abducting femur. The pressure was applied to the femur in the proximal direction in dorsal and lateral recumbency during abduction. The reduction of the luxated/subluxated femoral head was termed Ortolani positive. Clicking sounds are best perceived acoustically at the age of 16 to 32 weeks (4 to 8 months). Classification of the Barlow and Ortolani findings was based on those sounds, modified by Puerto et al. 1999 [26]. Depending on the clarity of the palpatory clunk or acoustic click, the tests were rated as (+), +, ++, and +++. The weakly positive tests were documented with (+). The diagnosis of (+) tests were very difficult and required high experience and they were recorded after several repetitions. Moderately positive clicking tests were recorded with +, hips with palpable clicking were recorded as ++, and hips with clearly palpable and/or audible clicking sounds were recorded as +++. If there was no hip joint instability, the maneuver was considered negative. In the case of a positive Ortolani sign but a non-palpable or not acoustically detectable Barlow sign, the result was classified as indefinable.

#### 2.3.3. Bardens Maneuver

The Bardens maneuver was only carried out in lateral recumbency. In a parallel positioning of the femur to the surface of the table, the pressure was applied from the medial to lateral section. In the case of instability of the hip joint, displacement of the trochanter major was palpated. The degree of mobility correlates with the degree of hip laxity. Two or less than 2 mm displacement was considered physiological displacement. Three to 4 mm displacement was considered a borderline case, 5 to 6 mm was considered marked or moderate hip laxity and above 6 mm was considered severe high-grade hip laxity. All orthopedic examinations were performed by the same investigator (B.V.).

### 2.4. Goniometric Assessments

Measurements of the RA and SA were performed with a manual goniometer in this study. The goniometer was located at the insertion of the pectineus muscle on the ileo-pectinal eminence of the pubis to avoid measurement errors. Measurements were repeated three times for each angle and mean values were recorded on each examination date. The RA was measured in dorsal recumbency during the abduction of the hip joint until dislocation of the femoral head, which was confirmed with a palpatory clunk or acoustic click, while the opposite femur was extended and kept parallel to the table. The SA was measured during limb adduction, at which point the femoral head can be subluxated. All examinations were performed by the same investigator (B.V.).

### 2.5. Radiographic Evaluation

The radiographic evaluation of all dogs followed the guidelines of the FCI in this study. The radiographs were taken in ventrodorsal projection with the extended femurs held parallel to each other and to the table surface. The Norberg angle was measured as described by Douglas 1970. Radiographs were exposed with 75–96 kV and 9 mAs with a film-focus distance of 90 cm in a storage phosphor screen/cassette system (Kodak Carestream, Health Inc., Rochester, NY, USA). All images were digitally stored and evaluated (dicomPACS View Version 6.0.2, 457, Bodmin, England and Oehm und Rehbein, Rostock, Germany).

**Figure 1:**
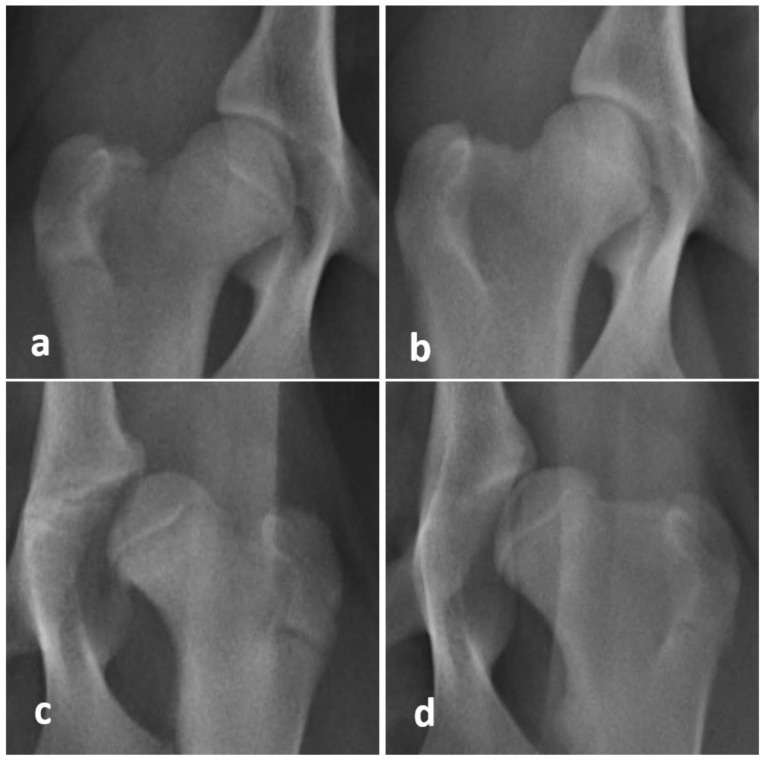
Magnification of an extended ventrodorsal view of the right and left hip joints of two Rottweilers: (**a**) A dog at the age of 31 weeks, and (**b**) the same dog at the age of 52 weeks. The joint was consistently graded as HD A for this dog. (**c**) Another dog at the age of 18 weeks, and (**d**) the same dog at the age of 30 weeks. The joint was consistently graded as HD E and unsurprisingly, did not improve with continuous age.

The dogs were graded from HD A to HD E according to FCI. Dogs with no hip dysplasia were categorized as category A, while dogs with nearly normal hip joints were rated as HD B. Dogs with mild, moderate, and severe grades of hip dysplasia were categorized as HD C, D, and E, respectively. All examinations were performed by the same investigator, who has been a certified scrutinizer for over 12 years (M.G.). The magnification of an extended ventrodorsal view of the right and left hip joints of two Rottweilers is shown in Figure 1. The final FCI grading was done at the age of 52 weeks (one year).

### 2.6. Statistical Analysis

Data analysis was performed using IBM SPSS statistics version 20 statistical software, and descriptive statistics were calculated for Ortolani, Barlow, Bardens, SA, RA, and FCI scores. The results with a p-value below 0.05 were considered significant. Cohen's kappa coefficient (κ) was used to evaluate the agreements between Ortolani findings in dorsal and lateral recumbency, as well as Barlow findings in dorsal and lateral recumbency (κ < 0 = poor agreement and κ = 1 high agreement). Furthermore, Pearson's chi-squared tests were performed to evaluate the difference between two sets of measurements (dorsal and lateral recumbency). One-way analysis of variance (ANOVA) with a Bonferroni post hoc test and Spearman’s correlation were used to analyze the correlation between RA and Ortolani findings. To investigate the correlation between RA/SA and the FCI score, as well as the correlation between Ortolani findings and FCI score, Spearman’s correlation, and ordinal regression were calculated.

## 3. Results

Thirty purebred Rottweilers of the Austrian Armed Forces were included in this study. Eleven dogs were female, and nineteen dogs were male. The M1 examination was performed on 28 dogs, with a mean body mass of 17 ± 2 kg (range: 12.5–22 kg). The M2 examination was performed on 30 dogs, with a mean body mass of 30 ± 2 kg (range: 25.1–33.2 kg), and the M3 examination was performed on 29 dogs, with a mean body mass of 35 ± 2 kg (range: 31–39 kg). The number of examined dogs varied during the study due to the inappropriate condition of single puppies (*n* = 2), such as non-related disease (enteritis), missing an examination date, and the death of one puppy. The recorded results are available in the Supplementary Materials.

### 3.1. Ortolani Findings

At M1, 17.9% of the hips had negative Ortolani results in dorsal recumbency, whereas 78.6% negative Ortolani results were recorded in lateral recumbency. At M2, 45.0% of the hips had negative Ortolani signs in dorsal recumbency, whereas, in lateral recumbency, 88.3% negative Ortolani signs were recorded. At M3, 63.8% negative Ortolani signs in dorsal recumbency and 87.9% negative Ortolani signs in lateral recumbency were recorded. The results of these Ortolani findings are shown in Table 1. In dorsal recumbency, 61.1% of the Ortolani tests were the same for both hips of the dogs, whereas, in lateral recumbency, 81.1% of the Ortolani tests were the same for both hips.

According to the Chi-square test, the number of negative Ortolani findings in dorsal recumbency was significantly lower than in lateral recumbency (*p* = 0.000). With one exception, all animals that had a negative Ortolani test in dorsal recumbency also had a negative Ortolani test in lateral recumbency. There was generally low to no agreement between the Ortolani test results in dorsal and lateral recumbences. Cohen's kappa coefficient for the first, second, and third examination date was κ = 0.104, κ = 0.084, and κ = 0.230, respectively. The results demonstrated that the Ortolani sign was more accurate in dorsal than lateral recumbency. False-negative Ortolani results were recorded in lateral recumbency, whereas the results of the same examinations were positive in dorsal recumbency. Therefore, examinations in dorsal recumbency had a high specificity. Furthermore, hips with mild to moderate Ortolani signs were differentiated only from each other in dorsal recumbency. There was a statistical difference between Ortolani in dorsal and lateral recumbency in our study. Therefore, we prefer the Ortolani test in dorsal recumbency. Our results showed that 91.7% of the Ortolani (+) or + dogs had an SA below 5°. This confirms that a small SA may indicate mild hip laxity. Therefore, no subluxation angle should be seen in sound hips.

### 3.2. Barlow Findings

The results of the Barlow negative tests in dorsal recumbency at M1, M2 and M3 were 17.9%, 45.0% and 65.5%, respectively. Furthermore, the negative Barlow tests in lateral recumbency at M1, M2 and M3 were 78.6%, 88.3% and 87.9% respectively. In dorsal recumbency, 53.4% of the Barlow tests were the same for both hips of the dogs, whereas, in lateral recumbency, 81.1% of the Barlow tests were the same for both hips. There was no agreement between the Barlow test results in dorsal and lateral recumbences. Cohen's kappa coefficient for the first, second, and third examination date was κ = 0.190, κ = 0.176, and κ = 0.272, respectively. The Barlow sign in lateral recumbency had high negative results, whereas, in dorsal recumbency, positive results were recorded for the same hips. Therefore, these negative results in lateral recumbency were considered to be false negatives. The difference between lateral and dorsal recumbency was significant in our study (*p* = 0.001). In general, the Barlow sign was often undetectable in both recumbencies. Therefore, the test had a low predictive value. The results of the Barlow findings are summarized in Table 2.

### 3.3. Bardens Findings

The negative Bardens signs at M1, M2 and M3 were 75.0%, 88.3%, and 94.8%, respectively. Eighty-seven percent of the Bardens tests were the same for both hips of the dogs. The results of the Bardens findings are listed in Table 3.

### 3.4. Subluxation Angle

The mean and standard deviation (range) of the measured SA at the first and second examination dates were 10.2° ± 7.8 (4.7°–15.7°) and 17.3° ± 4.7 (14°–20.7°), respectively. At the third examination date, 67.2% of the evaluated SA were undefinable, and the remaining 32.8% were negative. Therefore, no mean and standard deviation were recorded. The evaluation of the SA had low statistical relevance because of the high number of undefinable results, especially at the third examination date. Therefore, SA was excluded from statistical analysis.

### 3.5. Reduction Angle

The mean and standard deviation (range) of the measured RA at the first examination date were 21.5° ± 4.9 (11.0°–42.0°). At the second examination date, 21.1° ± 6.2 (11.3°–40.0°), and at the third examination date, 15.6° ± 7.6 (6.7°–36.7°) were recorded.

### 3.6. FCI Scoring

**Figure 2:**
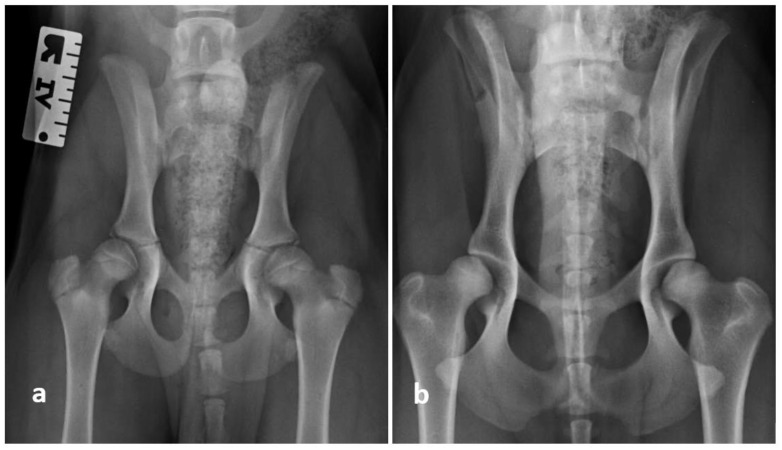
Magnification of an extended ventrodorsal view of both hip joints and the lumbosacral region of a Rottweiler (**a**) at the age of 18 weeks and (**b**) at the age of 52 weeks. The joint was graded as HD B/A (right/left side) on first examination. However, the final grading was HD C/B (right/left side). The dog also suffers from a lumbosacral transitional vertebra.

According to the FCI scoring method, at the third examination date (54 ± 1 weeks, N = 29 dogs), 57.1% (33 hips) of the dogs had no hip dysplasia and were classified as HD A, and 17.1% (10 hips) of the examined dogs had borderline hip dysplasia and were classified as HD B. Furthermore, 25.9% (15 hips) of the dogs had variable degrees of hip dysplasia and were classified as HD C (19%), HD D (4.3%), or HD E (4.3%). The magnification of an extended ventrodorsal view of both hip joints and the lumbosacral region of a Rottweiler at two different examination times are shown in Figure 2.

### 3.7. Correlation between Ortolani Findings and RA/SA

As reported above, due to the high number of not definable results and low statistical relevance, the correlation between the Ortolani test and SA was not calculated in this study. Evaluation of the Ortolani maneuver was performed together with the measurement of the RA in our clinic due to the nature of the two movements. An assessment of the correlation between the Ortolani test and RA showed a significant positive correlation between RA at M1 and the Ortolani findings as well in lateral as in dorsal recumbency (*p* = 0.001). At M2, a high correlation was only recorded between the Ortolani findings in lateral recumbency and RA (*p* = 0.003). At M3, a strong correlation was recorded between the Ortolani findings in lateral recumbency and RA (*p* = 0.001), whereas no significant correlation was recorded in dorsal recumbency (*p* = 0.087). The results of the correlation between the Ortolani test and RA are shown in Table 4.

### 3.8. Correlation between the FCI Score and RA/SA

A significant correlation was recorded between the RA and the results of the radiological studies according to the FCI guidelines at the second (*p* = 0.002) and third (*p* = 0.001) examination dates, whereas no significant correlation was found at the first examination date (*p* = 0.120). The correlation coefficient (r) increased with the age of the dogs from 0.2 at M1 to 0.5 at M3. The results of the correlation between the RA and FCI scoring method are shown in Table 5.

**Figure 3:**
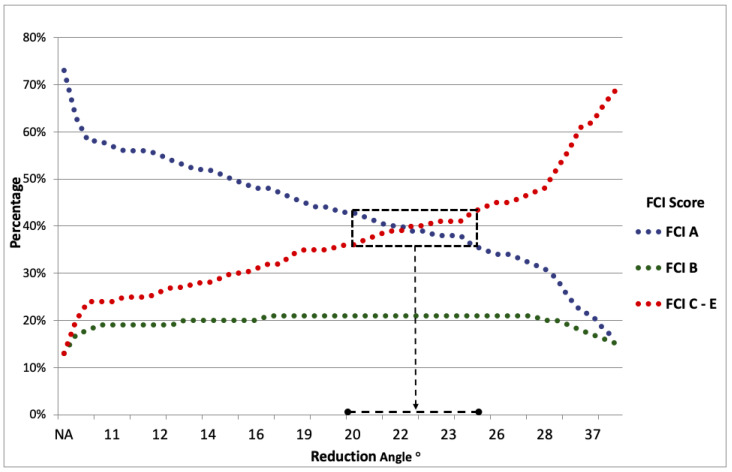
Ordinal regression of the reduction angle (RA) and probability graph for the development of hip laxity in dogs. The dotted blue, green, and red lines represent the probability for the development of category HD A, HD B, and HD C–E hips, respectively. The black square marks the zone in which the probability for category HD A and category HD C–E is between 36% and 43%. In this zone, the RA ranges from 20° to 25°. NA = not available.

The calculation of the ordinal regression showed a significant correlation between the measured RA and the final FCI score. Most of the dogs that were categorized as group A (no hip laxity) had low RA, whereas most of the dogs that were categorized as group C–E (mild to severe grades of hip laxity) had larger RA. Due to the low number of hips with severe grades of hip laxity, HD C, HD D, and HD E hips were combined in our study. As shown in Figure 3, prediction of the development of hip laxity for dogs with RA between 20° and 25° (area highlighted with black square marks) might be difficult as the category HD A and category HD C–E approximately overlap here.

### 3.9. Correlation between the FCI Score and Ortolani Findings in Dorsal Recumbency

**Figure 4:**
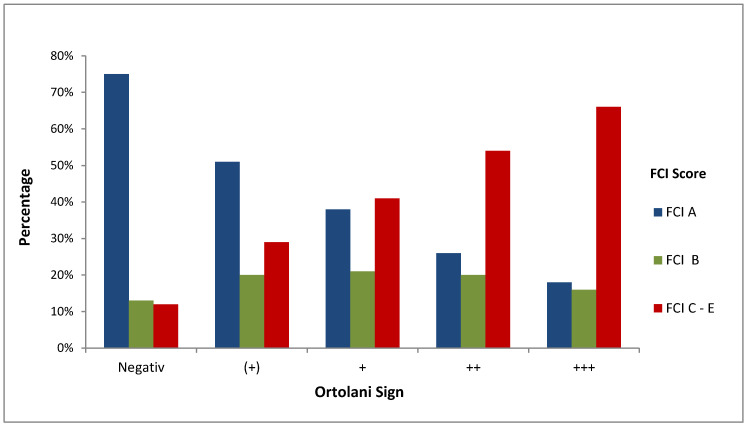
Ordinal regression of the Ortolani test and FCI scoring method in 30 Rottweiler puppies.

There is no significant correlation between the results of the Ortolani test in dorsal recumbency and the results of the FCI scoring method at the age of M1, whereas significant correlations at the ages of M2 (*p* = 0.001) and M3 (*p* = 0.000) were recorded. The calculation of the ordinal regression between the results of the Ortolani test and FCI scoring findings showed that 75% of the Ortolani negative dogs were classified as HD A, 13% of the Ortolani negative dogs were classified as HD B, and 12% of the Ortolani negative dogs were classified as HD C or more severe grades of hip laxity (Figure 4).

## 4. Discussion

This study was designed to evaluate the correlation between common clinical methods for the early diagnosis of hip joint laxity and radiographic findings according to the guidelines of the FCI in a closed cohort of Rottweilers. We hypothesized that there is a statistical difference between Ortolani findings in dorsal and lateral recumbency, as well as Barlow findings in dorsal and lateral recumbency. However, we hypothesized that there is a correlation between RA and Ortolani findings, between RA and FCI scores, between SA and FCI scores, and between Ortolani findings and FCI scores. The results confirmed all of our hypotheses except for SA.

All examinations were performed three times in this study. Four and eight months of age were considered as a predictor of hip dysplasia in the literature [27]. At four months of age, relative growth of the skeleton and muscle consistency are reached; before this age, passive laxity due to the undeveloped muscles and skeleton may affect the measurements. Therefore, the first examination time (M1) was 20 ± 2 weeks in this study. At eight months of age, the main part of skeletal maturity is reached. Therefore, we performed the second round of the examinations (M2) at 35 ± 2 weeks. The third examination (M3) was performed at approximately one year at the age of 54 ± 1 weeks, according to the recommended FCI guidelines.

In our study, at the first examination time, the number of the Barlow negative and Ortolani negative hips was the same, and the results of the Ortolani findings in dorsal recumbency were definable but at the same examination time, 41.1% of the Barlow findings in dorsal recumbency were indefinable (positive Ortolani sign but not detectable Barlow sign). The number of Bardens negative hips at the three examination times was almost the same as the results of the Ortolani negative and Barlow negative hips in lateral recumbency. The comparison of Bardens and the other two orthopedic examinations was only conducted in lateral recumbency as the Bardens test is only performed in lateral recumbency. The ideal age for performing the Bardens test is reported to be between 8 and 9 weeks, and an 83% accuracy was reported for this method [20]. Performing and evaluating the Bardens test requires a high level of competence and experience [28]. In our study, the results of the Bardens tests at 20 ± 2 weeks of age were similar to those of Ortolani and Barlow tests. Ortolani positive hips could be classified as the different groups according to the severity of laxity, as reported before, while the hips with positive Bardens results were not classified. The hips which were not Barlow negative were classified as positive or indefinable, which seems to be more accurate than Bardens results, which were all classified as positive or negative.

The relationship between the Ortolani test and RA illustrates the importance and validity of both early diagnostic methods. The current study showed that the Ortolani sign correlated significantly with RA. This correlation increases with the grade of the Ortolani test and the age of the dogs. As reported before the results of Ortolani tests in dorsal recumbency were more accurate than those in lateral recumbency in our study. Despite these findings, at the second and third examination times, the correlation between RA and the Ortolani test was only statistically significant in lateral recumbency. At the second examination time, the correlation between RA and the Ortolani test in dorsal recumbency was just below the level of significance (*p* = 0.063), and at the third examination time, the correlation between RA and the Ortolani test in dorsal recumbency was even worse (*p* = 0.087). Further studies are recommended to assess the relationship between RA and the Ortolani maneuver in lateral and dorsal recumbency.

There was a positive correlation between measured the RA and FCI scores in this study. This correlation increased with the age of the animals. The same results were reported for RA and other radiographic methods, such as the distraction index and dorsal acetabular rim angle, in the literature [19]. As reported, a high number of indefinable results (67.2%) were reported for the measurements of SA in our study, especially at the third examination time. Therefore, due to the low statistical validity, the SA was excluded from statistical analysis and the correlation between SA and the FCI score was not investigated.

A positive correlation was recorded between the Ortolani sign in dorsal recumbency and the FCI scoring mode at the second and third examination dates (M2 and M3), but no correlation was recorded at the first examination date (M1). Therefore, a significant correlation was only observed in young and adult dogs. One of the reasons for this may be false-positive Ortolani results at this age. As reported previously, at four months of age, relative growth of the skeleton and muscle consistency are reached, but in comparison with eight and twelve months of age, this consistency may not be optimal. On the other hand, from 8 to 12 weeks of age, the degree of laxity may increase because of swelling and expansion of the joint capsule [29,30].

In a study performed in 2018 [29] on 306 large dog breeds, such as Labrador retrievers, golden retrievers, German shepherds, giant poodles and mixed breeds at 4, 6, and 12 months of age, a significant increase was reported for DI from 4 to 6 months of age, but the values decreased again from 6 months of age. At 12 months of age, the DI values were significantly lower than at 4 months of age. It should be noted that at older ages with severe grades of laxity, an orthopedic examination such as the Ortolani test might be negative because of the fibrosis of the joint capsule. Therefore, a negative Ortolani result does not guarantee a healthy hip joint [30].

As reported in the literature, an early diagnosis of hip laxity with orthopedic examinations is possible from 16 to 20 weeks of age in large breeds such as greyhounds, Labrador retrievers, and Irish setters [31]. Contrary to these findings, in our study, the final FCI score could be predicted from the second examination time (35 ± 2 weeks). One reason for this difference might be the small sample size. Therefore, the authors suggest further investigations with a larger population. Another reason might be a breed difference, as only Rottweilers were examined in this study. To the best of the authors' knowledge, no study has previously investigated the early diagnosis of hip laxity in purebred Rottweilers.

Corfield et al. 2007 [32] indicated that 22.7% of the Ortolani negative hips in Labrador retrievers at 4 months of age did not show osteoarthritis at 12 months of age, and of the remaining 77.3% Ortolani positive hips, about 41% exhibited osteoarthritis by 12 months of age. The presence of osteoarthritis at the age of 12 months was not correlated with the measured RA in this study, so the RA alone was unreliable for predicting the status of osteoarthritis at 12 months of age [32].

Gatineau et al. 2012 [19] investigated the Ortolani sign at 6 months of age in large dog breeds (Labrador retrievers, Bernese mountain dogs, Labrador retriever–Bernese mountain mixed breed dogs) to predict the occurrence of coxofemoral osteoarthritis at 24 months of age, and reported a 100% sensitivity, and 41% specificity for this method. None of the Ortolani negative hips at the age of 6 months showed a radiographic sign of osteoarthritis at the age of 24 months. Only 43% of the Ortolani-positive hips developed osteoarthritis at 24 months of age, whereas 57% were free of osteoarthritis [19].

In a study performed by Ginja et al. [15] on Estrela mountain dogs, a 92% sensitivity was reported for the Ortolani test, though the specificity of the Ortolani test was lower than that of PennHIP and FCI methods in this study. These findings confirmed the results reported by Vezzoni et al. [21]: A negative Ortolani sign among young puppies does not exclude the possibility of a positive Ortolani sign at later ages. The number of false-negative tests decreased in older dogs (Italian cane corsos, Labrador retrievers, German shepherds, golden retrievers, Rottweilers, Bernese mountain dogs, Newfoundlands, border collies, Dobermans, etc.), so the specificity of the Ortolani test was enhanced with the age of the dogs [21]. However, the sensitivity decreased with the age of the dogs, as four puppies with a positive Ortolani sign at the age of 17–28 weeks had normal hips after skeletal maturity was reached. According to the authors of the mentioned study, the prediction of hip dysplasia according to the FCI scoring mode based on RA is rather possible, but not sufficient as a single value [21].

Taroni et al. [33] reported that the DI at the age of 4 months in large breed dogs such as Labradors, golden retrievers, German shepherds, and large mixed breed dogs could not properly predict the FCI score at 12 months of age. These results are similar to our results reported for prediction of the FCI score at 4 months of age by the Ortolani maneuver. In a study performed in 2020 [34] on mostly large breeds (German shepherds, Labrador retrievers, golden retrievers, Doberman pinschers, etc.), a better intraclass correlation coefficient was recorded for NA in comparison with the DI. Furthermore, the FCI scoring method was reported to be marginally more reliable than the Swiss scoring method.

Our results are in contrast to the results reported by Gulanber et al. [35], who found no significant correlation between the Ortolani findings in 122 large breed dogs (golden retrievers, Labrador retrievers, German shepherds, Turkish shepherd dogs, etc.) at 3 and 9 months of age and the results of the FCI scoring method at 18 months of age. In this study, the number of dogs with mild to severe grades of hip dysplasia at 18 months of age was higher than the number of Ortolani positive dogs at 3 and 9 months of age [35]. One of the reasons for this may be the severity of laxity between different dog breeds. As reported by Adams et al. [36], the Bardens maneuver was only a significant predictor of hip dysplasia only in golden retrievers, whereas it was not a reliable predictor for Labrador retrievers and golden retriever puppies (6 to 9 weeks of age); however, the Ortolani maneuver was not a significant predictor of hip dysplasia in this study [36].

Puerto et al. [26] reported a significant correlation between the Ortolani sign and DI, whereas a low to moderate correlation between the Ortolani sign and OFA hip scoring method was recorded in this study. The OFA hip scoring method is used in the USA and Canada and based on evaluation of the shape and form of the femoral head and acetabulum, acetabulum coverage by femoral head, status of the joint space, and arthritic changes [24]. In this system, the dogs are divided into two groups. Normal hips with excellent, good, or fair score, and dysplastic hips with mild, moderate, or severe dysplasia [24]. Based on this study, degenerative articular changes, such as thickening of the joint capsule, may cause a negative Ortolani sign (false negative) in comparison with dogs without osteoarthritis. Therefore, in the case of a negative Ortolani sign, it is not possible to immediately diagnose a sound joint. Conversely, a positive Ortolani sign always suggests a hip joint laxity [26]. Therefore, a combination of palpatory and radiographic techniques is recommended as an early evaluation method. The results of these studies showed that the Ortolani sign provides a good indication for the development of HD A or HD C (or worse), while the prediction of HD B is difficult. The same findings were applied to the RA. According to the literature, it is important to use a combination of techniques to early diagnose hip laxity [26]. Due to the importance of the heredity and genetic background in this disease (OMIA 000473-9615), the early use of proper diagnostic methods is important for identifying the dysplastic dogs and preventing breeding with these animals [3,21,37]. The incidence of the CHD is reported to be mostly in large breed dogs such as golden retrievers, German shepherds, Labrador retrievers, and Rottweilers [29,30,31,32,33,35,38,39]. On the other hand, small breeds such as greyhounds and borzois have low risk of CHD [7,40]. A retrospective study on five large breeds between 1995 and 2016 in Switzerland showed that the prevalence of CHD decreased effectively in golden retrievers, Labrador retrievers, flat-coated retrievers, Bernese mountain dogs, and German shepherds due to the prevention of the breeding of HD D and HD E dogs [41]. As stated in the results, RA and the Ortolani test are two recommendable methods for the early diagnosis of hip laxity. Moreover, the SA could provide additional useful information. Despite the study by Adams et al. [36], the Bardens and Barlow tests were not as significant as the Ortolani test in our study. These two methods can only be used to differentiate the presence of hip laxity, and they are not suitable for interpreting the severity of the laxity. We were faced with several study limitations in this study, such as a small sample size and indefinable SA values. Therefore, we suggest a larger population for upcoming studies.

## 5. Conclusions

In conclusion, an early diagnosis of hip joint laxity and prediction of FCI scores from the ages of 35 ± 2 weeks by means of RA and the Ortolani maneuver is possible in Rottweilers. In addition, the Ortolani maneuver in dorsal recumbency is preferable to lateral recumbency due to the false-negative results in lateral recumbency; however, the Ortolani test was more accurate than Barlow and Bardens tests in this study. To minimize the errors during examinations, particular attention must be paid to the quality of the radiographs and radiographic positioning, as well as proper orthopedic examinations.

## Figures and Tables

**Table 1:** Descriptive statistics of the Ortolani tests reported for each examination date.

Examination Date ^1^	Recumbency	N (Dogs/Hips)	Frequency (Percentage) ^2^				
			−	(+)	+	++	+++
M1	Dorsal	28/56	10 (17.9%)	24 (42.9%)	17 (30.4%)	5 (8.9%)	0 (0%)
M1	Lateral	28/56	44 (78.6%)	3 (5.4%)	7 (12.5%)	1 (1.8%)	1 (1.8%)
M2	Dorsal	30/60	27 (45.0%)	12 (20.0%)	15 (25.0%)	4 (6.7%)	2 (3.3%)
M2	Lateral	30/60	53 (88.3%)	3 (5.0%)	2 (3.3%)	2 (3.3%)	0 (0%)
M3	Dorsal	29/58	37 (63.8%)	8 (13.8%)	7 (12.1%)	5 (8.6%)	1 (1.7%)
M3	Lateral	29/58	51 (87.9%)	3 (5.2%)	3 (5.2%)	0 (0%)	1 (1.7%)

^1^ M1: 20 ± 2 weeks, M2: 35 ± 2 weeks, and M3: 54 ± 1 weeks. ^2^ − = negative results (not palpable), (+) = moderate positive, + = barely noticeable clicking, ++ = hips with palpable clicking, +++ = hips with a clear palpable or audible clicking sound.

**Table 2:** Descriptive statistics of Barlow tests reported for each examination date.

Examination Date ^1^	Recumbency	N (Dogs/Hips)	Frequency (Percentage) ^2^		
			−	+	Not definable
M1	Dorsal	28/56	10 (17.9%)	23 (41.1%)	23 (41.1%)
M1	Lateral	28/56	44 (78.6%)	9 (16.1%)	3 (5.4%)
M2	Dorsal	30/60	27 (45.0%)	21 (35.0%)	12 (20.0%)
M2	Lateral	30/60	53 (88.3%)	5 (8.3%)	2 (3.3%)
M3	Dorsal	29/58	38 (65.5%)	12 (20.7%)	8 (13.8%)
M3	Lateral	29/58	51 (87.9%)	4 (6.9%)	3 (5.2%)

^1^ M1: 20 ± 2 weeks, M2: 35 ± 2 weeks, and M3: 54 ± 1 weeks. ^2^ Negative = negative results, Positive = positive results, Not definable = positive Ortolani sign but a non-palpable or acoustically detectable Barlow sign.

**Table 3:** Descriptive statistics of the Bardens tests reported for each examination date.

Examination Date ^1^	Recumbency	N (Dogs/Hips)	Frequency (Percentage) ^2^	
			−	+
M1	Lateral	28/56	42 (75.0%)	14 (25.0%)
M2	Lateral	30/60	53 (88.3%)	7 (11.7%)
M3	Lateral	29/58	55 (94.8%)	3 (5.2%)

^1^ M1: 20 ± 2 weeks, M2: 35 ± 2 weeks, and M3: 54 ± 1 weeks. ^2^ Negative = negative results, Positive = positive results.

**Table 4:** Correlation coefficients (r) and *p*-values of the Ortolani test with the reduction angle at three examination times.

Examination Date ^1^	Recumbency	r	*p*-Value
M1	Dorsal	0.564	0.001
M1	Lateral	0.473	0.001
M2	Dorsal	0.327	0.063
M2	Lateral	0.495	0.003
M3	Dorsal	0.392	0.087
M3	Lateral	0.657	0.001

^1^ M1: 20 ± 2 weeks, M2: 35 ± 2 weeks, and M3: 54 ± 1 weeks.

**Table 5:** Correlation coefficients (r) and *p*-values between the reduction angle and results of the Fédération Cynologique Internationale (FCI) scoring method.

Examination Date ^1^	r	*p*-Value
M1	0.214	0.120
M2	0.393	0.002
M3	0.527	0.001

^1^ M1: 20 ± 2 weeks, M2: 35 ± 2 weeks, and M3: 54 ± 1 weeks.

## Data Availability

The data presented in this study are available in supplementary material.

## Supplementary Materials

The following are available online at https://www.mdpi.com/2076-2615/11/2/416/s1, File S1: The description of results of the different examinations for all dogs.
