# Supplementary material for: Early Diagnosis of Canine Hip Laxity: Correlation between Clinical Orthopedic Examinations and the FCI Scoring Method in a Closed Cohort of Rottweilers
Source: Animals (Basel). 2021 Feb 6;11(2):416. doi: 10.3390/ani11020416 (PMC7915998; doi:10.3390/ani11020416)
Supplement: Supplementary file 1 [file animals-11-00416-s001.pdf]

Dog number	Age (weeks)	Sex	Examination time	Bodyweight (kg)	Ortolani Dorsal Left	Ortolani Dorsal Right	Ortolani Lateral Left	Ortolani Lateral Right	Barlow Dorsal Left	Barlow Dorsal Right	Barlow Lateral Left	Barlow Lateral Right	Bardens Left	Bardens Right	SA (°) Left	SA (°) Right	RA (°) Left	RA (°) Right	FCI Left	FCI Right	Dog Number
1	22	Female	1	.	4	3	5	3	1	1	1	1	1	1	15.89	4.67	42.00	23.33	.	.	1
1	34	Female	2	29.00	5	5	4	4	1	1	1	1	1	1	20.22	14.00	40.00	36.00	.	.	1
1	52	Female	3	33.00	0	4	0	3	0	1	0	1	0	0	nd	nd	nd	32.67	E	E	1
2	22	Female	1	.	3	4	4	2	1	1	1	nd	1	1	0.00	0.00	20.67	27.33	.	.	2
2	34	Female	2	30.00	4	4	0	3	1	1	0	1	1	1	16.44	28.00	0.00	0.00	.	.	2
3	22	Female	1	18.50	2	2	0	0	1	1	0	0	0	0	0.00	0.00	21.33	26.00	.	.	3
3	34	Female	2	28.00	3	2	0	0	1	nd	0	0	0	0	13.11	0.00	13.33	14.00	.	.	3
3	52	Female	3	33.00	5	0	5	0	1	0	1	0	0	0	0.00	nd	36.67	nd	C	A	3
4	22	Male	1	19.50	3	2	0	0	1	nd	0	0	0	0	0.00	0.00	23.33	22.00	.	.	4
4	34	Male	2	30.00	3	3	2	3	1	1	1	1	0	0	16.78	nd	16.33	27.33	.	.	4
4	52	Male	3	35.00	0	0	0	0	0	0	0	0	0	0	nd	nd	nd	nd	A	A	4
5	22	Male	1	20.00	0	0	0	0	0	0	0	0	0	0	nd	nd	nd	nd	.	.	5
5	34	Male	2	28.00	2	0	0	0	nd	0	0	0	0	0	10.44	nd	11.33	nd	.	.	5
5	52	Male	3	34.00	0	0	0	0	0	0	0	0	0	0	nd	nd	nd	nd	A	B	5
6	22	Male	1	20.00	3	3	3	3	1	1	1	1	0	0	0.00	0.00	30.00	26.00	.	.	6
6	34	Male	2	28.00	0	0	0	0	0	0	0	0	0	0	nd	nd	nd	nd	.	.	6
6	52	Male	3	35.30	4	3	0	3	1	0	0	1	0	0	0.00	nd	19.33	20.67	C	C	6
7	22	Male	1	.	4	2	3	2	1	nd	1	nd	1	1	0.00	nd	22.00	25.33	.	.	7
7	34	Male	2	30.00	0	0	0	0	0	0	0	0	0	0	nd	nd	nd	nd	.	.	7
7	52	Male	3	38.50	0	0	0	0	0	0	0	0	0	0	nd	nd	nd	nd	A	A	7
8	22	Male	1	20.50	0	0	0	0	0	0	0	0	0	0	nd	nd	nd	nd	.	.	8
8	34	Male	2	30.00	0	0	0	0	0	0	0	0	0	0	nd	nd	nd	nd	.	.	8
8	52	Male	3	36.50	0	0	0	0	0	0	0	0	0	0	nd	nd	nd	nd	A	A	8
9	18	Female	1	15.50	2	3	2	3	nd	1	nd	1	0	1	0.00	0.00	20.67	26.67	.	.	9
9	34	Female	2	31.50	0	2	0	0	0	nd	0	0	0	0	nd	0.00	nd	21.67	.	.	9
9	52	Female	3	.	0	0	0	0	0	0	0	0	0	0	nd	nd	nd	nd	A	A	9
10	18	Male	1	14.50	2	3	0	0	nd	1	0	0	0	1	0.00	0.00	18.00	22.67	.	.	10
10	34	Male	2	33.20	0	0	0	0	0	0	0	0	0	0	nd	nd	nd	nd	.	.	10
10	52	Male	3	39.00	0	0	0	0	0	0	0	0	0	0	nd	nd	nd	nd	A	A	10
11	18	Female	1	12.50	2	3	0	3	nd	1	0	1	0	1	19.67	0.00	20.00	15.67	.	.	11
11	34	Female	2	27.00	2	2	0	0	nd	nd	0	0	0	0	21.56	0.00	21.67	22.67	.	.	11
11	52	Female	3	30.90	0	0	0	0	0	0	0	0	0	0	nd	nd	nd	nd	C	B	11
12	18	Male	1	14.00	2	3	0	0	nd	1	0	0	0	1	0.00	0.00	18.00	21.33	.	.	12
12	34	Male	2	31.70	0	0	0	0	0	0	0	0	0	0	nd	nd	nd	nd	.	.	12
12	52	Male	3	35.00	0	0	0	0	0	0	0	0	0	0	nd	nd	nd	nd	A	A	12
13	16	Male	1	16.70	0	0	0	0	0	0	0	0	0	0	nd	nd	nd	nd	.	.	13
13	39	Male	2	32.30	0	0	0	0	0	0	0	0	0	0	nd	nd	nd	nd	.	.	13
13	54	Male	3	36.30	0	0	0	0	0	0	0	0	0	0	nd	nd	nd	nd	B	B	13
14	16	Female	1	14.55	2	2	0	0	nd	nd	0	0	0	0	0.00	nd	13.67	19.00	.	.	14
14	38	Female	2	29.50	0	0	0	0	0	0	0	0	0	0	nd	nd	nd	nd	.	.	14
14	54	Female	3	31.50	0	0	0	0	0	0	0	0	0	0	nd	nd	nd	nd	C	B	14
15	16	Female	1	15.00	0	2	0	0	0	nd	0	0	0	0	nd	0.00	nd	11.50	.	.	15
15	39	Female	2	31.30	0	0	0	0	0	0	0	0	0	0	nd	nd	nd	nd	.	.	15
15	54	Female	3	32.00	0	0	0	0	0	0	0	0	0	0	nd	nd	nd	nd	A	A	15
16	16	Female	1	14.70	0	0	0	0	0	0	0	0	0	0	nd	nd	nd	nd	.	.	16
16	38	Female	2	28.80	0	0	2	0	0	0	nd	0	0	0	nd	nd	nd	nd	.	.	16
16	54	Female	3	32.00	2	0	2	0	nd	0	nd	0	0	0	0.00	nd	12.67	nd	B	A	16
17	16	Male	1	15.35	2	3	0	0	nd	1	0	0	0	0	0.00	0.00	11.00	19.00	.	.	17
17	39	Male	2	29.90	2	4	0	0	nd	1	0	0	0	0	0.00	0.00	22.00	23.33	.	.	17
17	54	Male	3	33.30	0	3	0	2	0	1	0	nd	0	0	nd	0.00	nd	10.67	A	B	17
18	31	Female	2	31.10	2	3	2	0	nd	1	nd	0	0	0	0.00	0.00	25.67	20.00	.	.	18
18	54	Female	3	33.00	2	2	0	0	nd	nd	0	0	0	0	0.00	0.00	18.67	14.67	B	C	18
19	20	Male	1	18.90	2	2	0	0	nd	nd	0	0	0	0	0.00	0.00	20.00	16.67	.	.	19
19	35	Male	2	30.80	3	3	0	0	1	1	0	0	0	1	26.44	0.00	27.33	20.00	.	.	19
19	55	Male	3	37.70	2	2	0	0	nd	nd	0	0	0	0	0.00	0.00	14.00	12.00	A	A	19
20	20	Male	1	16.00	3	3	0	0	1	1	0	0	0	0	0.00	0.00	19.33	20.00	.	.	20
20	35	Male	2	27.50	3	3	0	0	1	1	0	0	0	1	20.67	0.00	20.00	20.67	.	.	20
20	55	Male	3	34.20	2	3	0	0	nd	1	0	0	0	0	0.00	0.00	10.00	10.67	B	D	20
21	20	Male	1	16.70	2	2	0	0	nd	nd	0	0	0	0	0.00	0.00	21.33	19.67	.	.	21
21	35	Male	2	31.90	3	3	0	0	1	1	0	0	0	0	17.56	0.00	16.67	12.00	.	.	21
21	55	Male	3	38.30	3	3	0	0	1	1	0	0	0	0	0.00	0.00	6.67	10.67	A	A	21
22	20	Male	1	18.40	2	3	0	0	nd	1	0	0	0	0	nd	0.00	18.67	22.33	.	.	22
22	35	Male	2	31.10	3	2	0	0	1	nd	0	0	0	0	20.00	0.00	20.00	16.33	.	.	22
22	55	Male	3	38.00	0	3	0	0	0	1	0	0	0	0	nd	0.00	nd	10.33	A	A	22
23	20	Male	1	19.05	4	3	3	0	1	1	1	0	0	0	0.00	0.00	28.00	21.33	.	.	23
23	35	Male	2	31.00	3	2	0	0	1	nd	0	0	0	1	27.33	0.00	28.00	19.33	.	.	23
23	55	Male	3	37.00	4	3	2	0	1	1	nd	0	1	1	0.00	0.00	20.00	12.00	C	A	23
24	20	Male	1	17.20	3	2	0	0	1	nd	0	0	0	1	0.00	0.00	23.00	20.00	.	.	24
24	35	Male	2	28.60	3	3	0	0	1	1	0	0	0	0	22.78	0.00	23.33	20.67	.	.	24
24	55	Male	3	31.30	2	2	0	0	nd	nd	0	0	0	0	0.00	0.00	10.33	14.00	C	C	24
25	20	Male	1	17.70	2	2	0	0	nd	nd	0	0	0	0	0.00	0.00	20.67	24.67	.	.	25
25	35	Male	2	31.00	2	4	0	0	nd	1	0	0	0	0	18.22	0.00	18.67	21.33	.	.	25
25	55	Male	3	35.60	3	4	0	3	1	1	0	1	0	1	0.00	0.00	10.00	22.00	C	D	25
26	35	Male	2	25.70	0	0	0	0	0	0	0	0	0	0	nd	nd	nd	nd	.	.	26
26	55	Male	3	37.50	0	0	0	0	0	0	0	0	0	0	0.00	nd	nd	nd	A	A	26
27	20	Female	1	16.80	2	2	0	0	nd	nd	0	0	1	0	20.89	0.00	22.33	20.00	.	.	27
27	35	Female	2	28.50	2	2	0	0	nd	nd	0	0	0	0	nd	0.00	20.67	14.67	.	.	27
27	55	Female	3	32.70	0	0	0	0	0	0	0	0	0	0	0.00	nd	nd	nd	A	B	27
28	20	Male	1	16.80	2	2	0	0	nd	nd	0	0	0	1	nd	0.00	17.00	19.67	.	.	28
28	35	Male	2	29.00	0	0	0	0	0	0	0	0	0	0	nd	nd	nd	nd	.	.	28
28	55	Male	3	33.00	0	0	0	0	0	0	0	0	0	0	nd	nd	nd	nd	A	A	28
29	20	Male	1	15.80	0	2	0	0	0	nd	0	0	0	0	nd	0.00	nd	22.67	.	.	29
29	35	Male	2	29.00	0	0	0	0	0	0	0	0	0	0	nd	nd	nd	nd	.	.	29
29	55	Male	3	34.00	0	0	0	0	0	0	0	0	0	0	0.00	nd	nd	nd	A	A	29
30	20	Female	1	15.40	3	3	0	0	1	1	0	0	0	1	nd	0.00	23.00	21.33	.	.	30
30	35	Female	2	25.10	0	3	0	0	0	1	0	0	0	0	nd	0.00	nd	18.00	.	.	30
30	55	Female	3	31.00	0	0	0	0	0	0	0	0	0	0	nd	nd	nd	nd	A	C	30

Ortolani:

0 : Negative
2: (+)
3: +
4: ++
5: +++

Barlow & Bardens:

0: Negative
1: +

nd: not definable
